# Evaluation of single cell oil (SCO) from a tropical marine yeast *Yarrowia lipolytica* NCIM 3589 as a potential feedstock for biodiesel

**DOI:** 10.1186/2191-0855-2-36

**Published:** 2012-07-19

**Authors:** Gouri Katre, Chirantan Joshi, Mahesh Khot, Smita Zinjarde, Ameeta RaviKumar

**Affiliations:** 1 Institute of Bioinformatics and Biotechnology, University of Pune, Ganeshkhind, Pune, 411 007, India; 2 Department of Biotechnology, Manipal Institute of Technology, Manipal, 576 104, India

**Keywords:** *Y. lipolytica*, Single cell oil, Fatty acid methyl ester, Biodiesel, WCO

## Abstract

Single cell oils (SCOs) accumulated by oleaginous yeasts have emerged as potential alternative feedstocks for biodiesel production. As lipid accumulation is species and substrate specific, selection of an appropriate strain is critical. Five strains of *Y. lipolytica*, a known model oleaginous yeast, were investigated to explore their potential for biodiesel production when grown on glucose and inexpensive wastes. All the strains were found to accumulate > 20% (w/w) of their dry cell mass as lipids with neutral lipid as the major fraction when grown on glucose and on wastes such as waste cooking oil (WCO), waste motor oil (WMO). However, amongst them, *Y. lipolytica* NCIM 3589, a tropical marine yeast, exhibited a maximal lipid/biomass coefficient, Y_L/X_ on 30 g L^-1^ glucose (0.29 g g^-1^) and on 100 g L^-1^ WCO (0.43 g g^-1^) with a high content of saturated and monounsaturated fatty acids similar to conventional vegetable oils used for biodiesel production. The experimentally determined and predicted biodiesel properties of strain 3589 when grown on glucose and WCO, such as density (0.81 and 1.04 g cm^-3^), viscosity (4.44 and 3.6 mm^2^ s^-1^), SN (190.81 and 256), IV (65.7 and 37.8) and CN (56.6 and 50.8) are reported for the first time for *Y. lipolytica* and correlate well with specified standards. Thus, the SCO of oleaginous tropical marine yeast *Y. lipolytica* NCIM 3589 could be used as a potential feedstock for biodiesel production.

## Introduction

The widespread use of fossil fuels such as petroleum, coal and natural gas, due to the high energy demand in today’s industrial world has led to problems of resource scarcity and environmental pollution. Fossil fuels have an additional disadvantage that they are not renewable. Biofuels as alternatives for petroleum fuel have generated great interest in recent years. Amongst them biodiesel from plant, algal and microbial sources seems to hold a partial solution to the ever increasing demand for energy, since their cell or biomass is renewable. General advantages of biodiesel include biodegradability, higher flash point, reduction in exhaust emissions, miscibility in all ratios with petrodiesel, compatibility with the existing fuel distribution infrastructure and inherent lubricity (Knothe 2008).

The use of microbes as feedstock for biodiesel has advantages such as their short life cycle, requires less labor and lower land resources, are easier to scale up, and are less affected by venue, season or climate (Li et al. 2008). Oleaginous microbes (bacteria, fungi and micro algae) are known to accumulate lipids in the form of triacylglycerols (TAGs) (Ratledge and Wynn 2002). The production of microbial lipids or Single Cell Oils (SCOs) has evoked considerable attention during the past decade since these SCOs can be used as a potential feedstock for the production of biodiesel (Meng et al. 2009; Kosa and Ragauskas 2011). Ramos et al. (2009) have shown that the biodiesel quality depends upon the fatty acid composition of the oil feedstock. For an oleaginous microbe to be considered as a suitable feedstock for biodiesel, the total lipid content (> 20%) and the type of fatty acids (long chain saturated and/or monounsaturated fatty acids) are important criteria. Lipid content and fatty acid composition of SCOs varies in response to environmental factors such as type of carbon source, pH, temperature and is species and strain-specific (Subramaniam et al. 2010; Venkata Subhash and Venkata Mohan 2011). This is evident from the studies on the psychrophilic oleaginous yeast *Rhodotorula glacialis* wherein both glucose concentration and temperature influenced the composition and degree of unsaturation of fatty acids (Amaretti et al. 2010). It has also been reported that the energy capacity of the dry yeast cell mass depends on the total lipid content, wherein 64% of the lipid content corresponded to 73% of the energy value of dry biomass (Minkevich et al. 2010). Consequently, since the accumulation of lipids by oleaginous yeasts varies, not all oleaginous yeasts can be used as a feedstock for biodiesel production. Therefore, careful selection of the oleaginous strains of the microbial species and characterization of lipid composition need to be performed to ascertain their suitability for biodiesel production.

Among the oleaginous yeasts, *Yarrowia lipolytica,* an unconventional microbe, has been extensively studied and is often isolated from lipid and hydrocarbon rich habitats, such as dairy products, polluted effluents and raw poultry. The potential biotechnological applications of *Y. lipolytica* using various environmental and industrial wastes have been discussed (Bankar et al. 2009). This yeast is known to degrade alkanes, fatty acids, fats and oil and is also a known model organism for lipid accumulation (Beopoulos et al. 2009; Fickers et al. 2005). Moreover, the unique ability of this yeast to efficiently use hydrophobic substrates makes this microorganism a prime candidate for use in the production of bio-oils (Beopoulos et al. 2009). Its hydrophobic substrate utilization and its metabolism directed towards lipid or SCO accumulation has been excellently reviewed by Fickers et al. (2005). Several technologies have been tried for SCO production by *Y. lipolytica* grown on various agro-industrial by-products or wastes and reasonably good cell growth and SCO production has been reported to occur on technical grade glycerol, animal fats, tallow, olive oil mill waste etc. with the major factions being saturated fatty acids (Papanikolaou and Aggelis 2010; Sarris et al. 2011). However, most of these studies have been directed towards production and utilization of these SCOs for various high value-added fats like cocoa butter, or using genetically modified strains for polyunsaturated fatty acids (PUFAs) having medical significance (Papanikolaou and Aggelis 2011). But, to date, hardly any reports from *Y. lipolytica* exist on either the use of SCO as feedstock for biodiesel or their physico-chemical characterization.

The present study investigates the biomass production and lipid accumulation potential of five different wild-type strains of *Y. lipolytica* grown on glucose. Further, utilization of some agro-industrial and other wastes for lipid accumulation by the selected strains were also studied. Lipid accumulation and the fatty acid composition in *Y. lipolytica* is known to depend on the substrate on which the cells are cultivated. These yeasts accumulate high levels of lipids when carbon is in excess and a key nutrient such as nitrogen or phosphorous is limiting (Ratledge and Wynn 2002). The accumulated lipids or SCOs get deposited as intracellular lipid bodies (LBs) which can easily be detected by the fluorescent probe, Nile red (Kimura et al. 2004). The cell mass of these yeast strains was evaluated for lipid content and their transesterified SCO profiles for biodiesel production as the type of fatty acids present are substrate dependent and important in ascertaining its appropriateness for biodiesel. Some physico-chemical properties of the biodiesel from the selected strain(s) were determined and compared with known international norms in order to ascertain its potential suitability as a fuel.

## Materials and methods

### Materials

Chloroform, methanol, acetone, formaldehyde, KH_2_PO_4_, Na_2_HPO_4_.12H_2_O, anhydrous Na_2_SO_4_, NaCl, KCl, and MgCl_2_ were of analytical grade and were purchased from Merck Ltd., Mumbai, India. 1,1,1-trichloroethane was obtained from National Chemicals, Vadodara, India. Potassium iodide and phenolphthalein were obtained from Fisher Scientific, Mumbai, India. Silicic acid, chromatography grade and Nile red were purchased from Sigma-Aldrich, Inc., USA. ‘Bead beater’ (Biospec Products, Inc., USA) was used for lysis of yeast cells and Kittiwake DIGI Biodiesel test kit (Kittiwake Developments Ltd., UK) was used for determination of Total Acid Number (TAN) and Free Fatty Acid (FFA) content. Iodine solution (Wijs) was purchased from Acros Organics, Belgium while alcoholic KOH solution was procured from Merck, Germany.

### Strains and growth conditions

Five strains of *Y. lipolytica* were obtained from The National Collection of Industrial Microorganisms (NCIM), National Chemical Laboratory, Pune, India. These were *Y. lipolytica* NCIM 3229 (NCYC 153), NCIM 3450, NCIM 3472 (ATCC 8661 and NCYC 825), NCIM 3589 and NCIM 3590 (NCYC 789 and MTCC 35), respectively. All strains were maintained on MGYP medium (g L^-1^), 3.0 malt extract; 3.0 yeast extract; 5.0 peptone and 10.0 dextrose, at 4°C. The medium used for cultivation of biomass was lipid accumulation medium (LAM), containing excess of carbon and limited in nitrogen, according to Suutari et al. (1993) and contained (g L^-1^), 30.0 glucose, 1.5 yeast extract, 0.5 NH_4_Cl, 5.0 Na_2_HPO_4_.12H_2_O, 7.0 KH_2_PO_4_, 1.5 MgSO_4_.7H_2_O, 0.1 CaCl_2_.2H_2_O, 0.01 ZnSO_4_.7H_2_O, 0.08 FeCl_3_.6H_2_O and (mg L^-1^) 0.1 CuSO_4_.5H_2_O, 0.1 Co [NO_3__2_.6H_2_O, 0.1 MnSO_4_.5H_2_O and pH adjusted to 5.5. An initial pre-inoculum of cells grown for 48 h in the abovementioned medium was used. The cells were washed with distilled water and count adjusted to 10^9^-10^10^ cells. These cells were inoculated in 100 ml of above mentioned media and incubated on a rotary incubator shaker (120 rpm) for 24, 48, 72 and 96 h at 30°C for all the four strains except *Y. lipolytica* NCIM 3590, which was grown at 20°C. For each sample, the experiments were carried out in triplicates.

### Nile red fluorescence

The fluorescent dye, Nile red was used to determine lipid accumulation ability of the five strains (Beopoulos et al. 2008; Mlickova et al. 2004). Cells were observed microscopically with an oil immersion objective using a Zeiss microscope (Axio Scope A1), equipped with a digital camera and a 465–495 nm excitation filter. ProgRes CapturePro 2.7 software (Jenoptik optical systems, USA) was used for recording the images.

### Extraction of yeast total lipids as SCO

The harvested cells were washed and lyophilized. This lyophilized dry biomass was weighed and used for extraction of total lipid using bead beater. The cells were added to the 20 ml chamber of the bead beater along with acid-washed glass beads (0.5 mm) to fill 3/4^th^ of the volume. Methanol (15 ml) was added to fill the chamber and a total of 10–12 cycles of 3 min each were run at 4°C to disrupt cells which was confirmed microscopically. Total lipids were extracted in chloroform: methanol (2:1, v/v) according to Schneiter and Daum (2006), the solvent vaccum evaporated and the residual lipid estimated gravimetrically.

### Lipid fractionation

The total yeast lipid (approximately 100 mg) was dissolved in 1 ml of chloroform: methanol (2:1, v/v) and loaded on a 25 mm × 100 mm silicic acid column (1 g silicic acid, activated by heating overnight at 110°C). The lipid was eluted from the column by sequential elution with 100 ml each of 1, 1, 1-trichloroethane, acetone, and methanol and the fractions collected. The solvent from each fraction was evaporated and the weight of the residual lipid determined. The fractions, in order of elution, were neutral lipids, glycolipids plus sphingolipids and phospholipids, respectively. The collected fractions were spotted on a silica gel F_254_ plate and developed according to Latge and De Bievre (1980). Appropriate authentic standards *viz*., tristearin, phosphatidylcholine and sphingomyelin were chromatographed on each plate. Experimental lipids were identified by comparing their *Rf* values with those of the standards. The fractions showing presence of neutral lipid were pooled to give total neutral lipid of the strain which was estimated gravimetrically.

### Transesterification of SCO and analysis of FAMEs

The SCO obtained was transesterified according to Leung et al. ( 2010). The reaction was carried out in a 50 ml round bottom flask kept in a thermostatic bath with a reflux condenser and a magnetic stirrer using a methanol to oil molar ratio of 60:1 and a catalyst (NaOH) concentration of 1.5-3 wt. % relative to SCO. The individual FAMEs in transesterified SCO (biodiesel) were detected using gas chromatography (GC) using CP-Sil88 column (50 m length, 0.25 μm ID) and Flame Ionization Detector (FID) as per the AOAC method (AOAC 2005). The resulting profile and retention times were compared with the standard (37 component FAME mix, Supelco, USA) and composition of the individual fatty acid methyl ester determined.

### Growth and SCO (total lipid) production of *Y. lipolytica* NCIM 3589 on varying initial glucose concentrations

The growth and total lipid production of the selected strain was checked on glucose concentration ranging from 10–100 g L^-1^ up to 96 h at 120 rpm at 30°C. All other conditions were the same as mentioned earlier. The residual glucose in the media was estimated using 3, 5-dinitrosalicylic acid (DNSA) according to Miller (1959).

### Preliminary screening of different wastes as substrates for SCO

Cheap and locally available substrates such as cheese whey (50%, v/v) and agro-residues such as groundnut shell, sugarcane bagasse, grape stalk, groundnut oil cake, copra meal, fruit and vegetable wastes like fruit peel (orange and banana), orange pulp and peapod were washed, dried, ground and passed through sieve of mesh size 1 mm and added as sole carbon and energy source (1% w/v) to the LAM medium. Fish and chicken wastes were autoclaved for 30 mins and added as minces (1% w/v) to the LAM medium. Prawn shell waste was de-mineralized by treatment with 1 N HCl (1:15 w/v) and de-proteinized by treatment with 3% NaOH (1:10 w/v), the residue washed, dried and added at 1% (w/v) in the LAM medium. Waste motor oil (WMO) and waste cooking oil (WCO) were also added as 1% (v/v) to the medium as described above. The biomass grown on WCO and WMO was made oil-free as per the protocol of Papanikolaou et al. ( 2010).

A pre-inoculum of the yeast cells was prepared as mentioned above and a cell count of 10^9^-10^10^ cells/50 ml was used to inoculate each flask. The flasks were incubated in a rotary incubator shaker (120 rpm) for 72 h at 30°C for all strains except 3590, which was incubated at 20°C.

### Growth and SCO (total lipid) production of *Y. lipolytica* NCIM 3589 and *Y. lipolytica* NCIM 3472 on varying concentrations of WCO

The growth and total lipid production of the selected strains was checked on WCO concentrations ranging from 10–100 g L^-1^ at 72 h (120 rpm, 30°C). The cells were made fat-free (Papanikolaou et al. 2010), the SCO extracted, transesterified and FAMEs were detected as mentioned earlier.

### Physico-chemical characterization of biodiesel properties of *Y. lipolytica* NCIM 3589 on glucose and WCO and *Y. lipolytica* NCIM 3472 on WCO

The physico-chemical properties of the biodiesel (FAME) from the strains selected by screening on glucose (NCIM 3589) and on WCO (NCIM 3589 and 3472) were evaluated. Density, kinematic viscosity, saponification number (SN), iodine value (IV) were determined experimentally as well as by using predictive models and mathematical equations for the transesterified SCOs. Density was determined gravimetrically at 25°C using a Pycnometer (10 ml). It was also predicted as per Kay’s mixing rule (Pratas et al. 2011) as follows:

(1) $$\rho =\sum_{}^{}c_{i}\rho_{i}$$

(where c_i_ and ρ_i_ denote concentration and density of individual component i, respectively). These values were obtained using the density of individual, pure FAME compounds from the database (Lapuerta et al. 2010).

The values for kinematic viscosity (40°C; mm^2^ s^-1^) were calculated by using the modified equation of Grunberg-Nissan as follows:

(2) $$v_{\mathrm{mix}}=\sum A_{c}\times v_{c}$$

in which ν_mix_ = the kinematic viscosity of the biodiesel sample (mixture of fatty acid alkyl esters), A_C_ = the relative amount (%/100) of the individual neat ester in the mixture (as determined by gas chromatography) and ν_C_ = the kinematic viscosity of the individual esters from database of FAMEs present in biodiesel (Knothe and Steidley 2011).

SN and IV were determined experimentally (AOAC 1975) and also calculated using the equations (3) and (4) as follows:

(3) $$SN=\sum \frac{\left(560\times A_{i}\right)}{MW_{i}}$$

(4) $$IV=\sum \frac{\left(254\times D\times A_{i}\right)}{MW_{i}}$$

where, A_i_ is the percentage, D is the number of double bonds and MW_i_ is the molecular mass of each fatty acid methyl ester (Azam et al. 2005; Gunstone et al. 2007)

Another fuel property, Higher Heating Value (HHV), a measure of the heat content of the oil was found to depend upon SN and IV values and was estimated using equation (5) (Demirbas 1998).

(5) $$HHV=49.43-\left[0.04(SN)+0.015(IV)\right]$$

Cetane Number (CN) was calculated using the multiple linear regression equation. (6) (Tong et al. 2011)

(6) $$CN=1.068\sum (CN_{i}W_{i})-6.747$$

where, CN_i_ represent reported CN of pure fatty acid methyl ester available in database (Tong et al. 2011) and W_i_ is the mass fraction of individual fatty ester component detected and quantified by GC-FID.

Water content of the sample, TAN and FFA were determined using the Kittiwake DIGI Biodiesel test kit while the copper strip corrosion test was carried out according to ASTM D130 test specifications.

### Statistical analysis

All values are means of three independent experiments. Statistical analyses were performed using SPSS 17 statistics software (SPSS Inc., Chicago, IL, USA). Means were compared and analyzed using either *t*-test or one-way analysis of variance (ANOVA) with Tukey HSD *post hoc* multiple comparison test. Differences were considered statistically significant for p < 0.05.

## Results

### Growth and SCO yields of *Y. lipolytica* strains on glucose

Lipid accumulation in oleaginous yeasts occurs as LBs when a nutrient in the medium e.g., nitrogen or phosphorous is limiting and carbon is in excess. The lipid yields, fatty acid composition and degree of unsaturation are affected depending on the type and concentration of carbon source (Granger et al. 1993; Ratledge and Wynn 2002; Amaretti et al. 2010). Therefore, we first investigated the effect of glucose, on cell growth, lipid yields and sugar consumption by different *Y. lipolytica* strains.

#### Determination of lipid accumulation by Nile red staining

**Figure 1:**
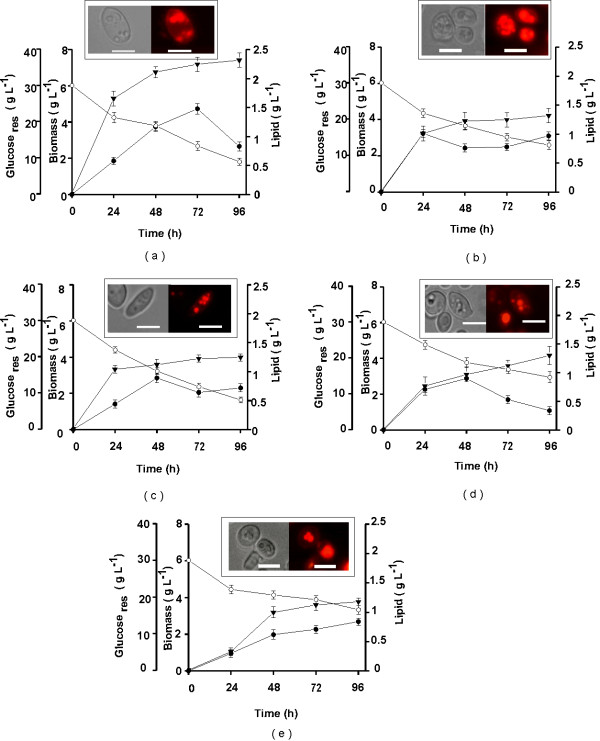
**Kinetics of biomass, residual glucose and cellular lipids of *Y. lipolytica *strains grown on glucose.** Strains (**a**) 3229, (**b**) 3450, (**c**) 3472, (**d**) 3589 and (**e**) 3590 were grown on 30 g L^-1^ glucose as mentioned in Materials and Methods. All values are represented as Mean ± SD, determined after 3 independent experiments. Biomass (g L^-1^) - ▾, Lipid (g L^-1^) - •, Glucose _res_ (g L^-1^) - ○. **Inset** In each graph light microscopy (left panel) and Nile red fluorescence microscopy (right panel) images of the respective *Y. lipolytica* strains under 100x oil immersion objective. Bar indicates 10 μm for **a**, **c** and **d** and 5 μm for **b** and **e**.

In the present study, all five strains of *Y. lipolytica* when grown in 30 g L ^-1^ glucose revealed a variable number of LBs which could be visualized by light microscopy of formaldehyde fixed cells or by fluorescence microscopy of cells stained with Nile red (Figure 1 insets). Variation in number and size of the LBs could be seen in all the strains grown in glucose. Therefore, all the five lipid accumulating strains *viz.*, NCIM 3229, NCIM 3450, NCIM 3472, NCIM 3589, and NCIM 3590 were further assessed for their total cellular lipid (SCO) content.

#### Time course for biomass production and total SCO yields

Figure 1 depicts the time course of biomass produced (X, g L^-1^), lipid yield (L, g L^-1^) and residual glucose concentrations (Glucose _res_, g L^-1^) against fermentation time in lipid accumulation medium containing 30 g L^-1^ glucose. Under these conditions, all the 5 strains showed an increase in biomass which varied from 3.77-7.42 g L^-1^ while the maximal lipid yield (L_max_, g L^-1^) varied from 0.84-1.48 g L^-1^ and were achieved in 24–96 h after incubation. The lipid/dry biomass yield coefficient (Y_L/X_, g g ^-1^) determined were 0.22 g g^-1^ in 96 h for strain 3229 (Figure 1a), 0.31 and 0.28 g g^-1^ in 24 h for strains 3450 and 3590 (Figure 1b, e), 0.25 and 0.29 g g^-1^ in 48 h for strains 3472 and 3589 (Figure 1c, d), respectively. Both, L_max_ and Y _L/X_ were found to decrease on prolonged incubation and were strain dependent. In all the strains under study, significant amounts of glucose (8.0-16.5 g L^-1^) remained unconsumed in the media even after 96 h of incubation. Thus, all the strains showed a tendency to degrade their storage lipids even though significant amounts of residual glucose remained in the medium. The total lipid content varied from 22-31% of the total dry weight of biomass and ascertained the oleaginous nature (> 20% lipid of the cellular weight) of all the five strains of *Y. lipolytica* tested.

#### Lipid fractionation

**Figure 2:**
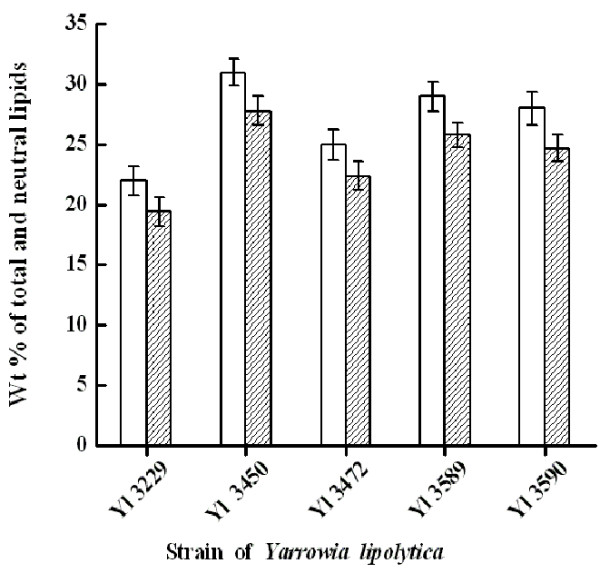
**Neutral lipid content of different strains of *Y. lipolytica.*** The data are expressed as mean ± standard deviation (n = 3). Mean values for total lipid and neutral lipid content (wt %) were determined as mentioned in Materials and Methods and did not differ significantly (p < 0.05). **Bar (empty): Total Lipids (wt %)and Bar (oblique line): Neutral lipids (wt %).**

The extracted SCOs from the five yeast strains grown on glucose were fractionated by silicic acid column chromatography to determine the individual lipid fractions. In all cases, regardless of the quantity of lipid accumulated inside the yeast, the neutral lipid fraction was the most abundant, whereas glycolipid plus sphingolipid and phosopholipid fractions were found in noticeably lower quantities (< 10%). The neutral lipid fraction includes triacylglycerols (TAGs), one of the key components desirable for biodiesel and the neutral lipid contents of all the strains were found to lie between 88-89% of the total lipid or SCO content (Figure 2). Thus, the cell mass of all 5 strains contained a high content of SCO with neutral lipid as the major fraction which is the desirable lipid type for biodiesel

#### Fatty acid profiles of transesterified SCOs

The SCOs obtained from the yeast strains were subjected to alkali catalyzed trans-esterification to give fatty acid methyl esters (FAME) or biodiesel. The resulting FAME profile and retention times of each methyl ester were compared with the authentic standard (37 component FAME mix, Supelco, USA) by GC-FID, the composition of FAME determined and profiles evaluated for their biodiesel suitability (Table 1).

**Table 1:** **Fatty acid methyl ester profiles of *Y. lipolytica *strains on glucose**

**% of fatty acid methyl ester**	**NCIM 3229**	**NCIM 3450**	**NCIM 3472**	**NCIM 3589**	**NCIM 3590**
Caprylic acid (C8:0)	ND	ND	ND	ND	0.4
Lauric acid (C12:0)	ND	ND	ND	ND	4.8
Tridecanoic acid (C13:0)	58.1	4.9	ND	ND	ND
Myristic acid (C14:0)	ND	ND	ND	0.4	3.9
Pentadecanoic acid (C15:0)	12.5	ND	ND	0.1	34.4
Palmitic acid (C16:0)	ND	ND	ND	24.1	2.8
Heptadecanoic acid (C17:0)	ND	ND	ND	ND	0.4
Stearic acid (C18:0)	ND	ND	ND	7.7	4.6
Arachidic acid (C20:0)	ND	ND	4.8	0.4	ND
Heneicosanoic acid (C21:0)	ND	ND	ND	ND	7.4
Behenic acid (C22:0)	ND	ND	12.3	0.4	ND
Lignoceric acid (C24:0)	ND	ND	ND	1.5	ND
Palmitoleic acid C16:1)	ND	ND	ND	11	1.6
Oleic acid (C18:1n9c)	ND	ND	ND	38.6	3.5
cis-10-pentadecanoic acid (C15:1)	ND	13.5	ND	ND	ND
cis-11Eicosanoic acid (C20:1)	ND	ND	ND	0.1	ND
Erucic acid (C22:1n9)	ND	ND	ND	ND	9.2
cis-10-Heptadecanoic acid (C17:1)	0.6	9.3	ND	0.4	ND
Linoleic acid (C18:2n6c)	ND	ND	ND	14.6	2.7
Linolenic acid (C18:2nc)	ND	ND	ND	0.1	ND
cis-11,14-Eicosadienoic acid (C20:2)	ND	ND	7.2	ND	ND
cis-8,11,14-Eicosatrienoic acid (C20:3n3)	3.7	10.3	ND	0.2	ND
Arachidonic acid (C20:4n6)	5.0	19.4	21.7	ND	9.8
cis-13,16-Docosadienoic acid (C22:2)	5.5	16.8	16.7	ND	ND
cis-5,8,11,14,17-Eicosapentanoic acid (C20:5n3)	6.4	16.7	19.2	0.2	9.1
cis-4,7,10,13,16,19-Docosahexanoic acid (C22:6n3)	8.2	9.2	17.6	ND	ND
Elaidic acid methyl ester(C18:1n9t)	ND	ND	0.5	ND	ND
Total of trans fat	ND	ND	0.5	ND	ND
Total of fatty acids: Saturated	70.6	4.9	17.1	34.6	64.1
Total of fatty acids: Monounsaturated	0.6	22.8	ND	50.1	14.3
Total of fatty acids: Polyunsaturated	28.8	72.4	82.4	15.1	21.6
Total of fatty acids	99.4	100.1	100	99.8	100

ND: Not detected.

Values are means of three independent sets of experiments.

The content of total saturated fatty acid (SFA) was highest in 3229 (70.6%) followed by 3590 (64.1%), 3589 (34.6%), 3472 (17.1%) and 3450 (4.9%) when grown on glucose. Of the total SFAs, strains 3229 and 3450 contained 58.1% and 4.9% of odd-chain fatty acid - C13:0 while strains 3590 and 3229 showed 34% and 12.5% of C15:0. Palmitic acid (C16:0) was highest in strain 3589 (24.1%) while stearic acid (C18:0) was present at 7.7% and 4.6% in 3589 and 3590. Strain 3472 contained 4.8% arachidic acid (C20:0) and behenic acid C22:0 (12.3%) while 7.4% of eicosanoic (C21:0) was present in 3590. Thus, only the strains 3589 and 3590 contained good amounts C16:0 and C18:0 SFAs required for biodiesel.

The total content of monounsaturated fatty acids, (MUFA) the desirable fatty acids for biodiesel was the highest in 3589 (50.1%). Lesser amounts were present in 3450 (22.8%) and 3590 (14.3%) while negligible amounts were present in 3472 and 3229. Palmitoleic acid (C16:1) and oleic acid (C18:1) were highest in 3589 (11% and 38.6%, respectively). Strain 3450 contained 13.5% cis-10-pentadecanoic acid (C15:1) while erucic acid, C22:1 (9.2%) was present in 3590 and cis-10-heptadecanoic acid (C17:1) was present at 9.3% in 3450. Thus, the long chain MUFAs, C16:1 and C18:1, required for good quality biodiesel were present in high amounts only in strain 3589.

The total polyunsaturated fatty acids (PUFAs) were highest in 3472 (82.4%) followed by 3450 (72.4%) 3229 (28.8%), 3590 (21.6%) and the least in 3589 (15.1%). PUFAs containing  ≥ 4 double bonds are not desirable for biodiesel (1% max, according to EN 14214). PUFAs like arachidonic acid (C20:4), eicosapentanoic acid (C20:5) and docosahexanoic acid (C22:6) were present in higher amounts in all four strains except 3589 (19.6% in 3229, 45.3% in 3450, 58.5% in 3472, 0.2% in 3589 and 18.9% in 3590, respectively) making the SCOs of these strains (except strain 3589) unsuitable for biodiesel.

Hence, depending on the neutral lipid content, fatty acid profile and based on the evaluation put forth by Ramos et al. (2009), strain 3589 which demonstrated a good neutral lipid content, a high SFA (34.6%) and maximal MUFA content (50.1%) and comprising of palmitic (C16:0), stearic (C18:0), palmitoleleic (C16:1) and oleic acid (C18:1) and low PUFA content (≥ 4 double bonds, 0.2%) was found to be the most suitable for biodiesel production.

#### Effect of initial glucose concentration on SCO production

**Figure 3:**
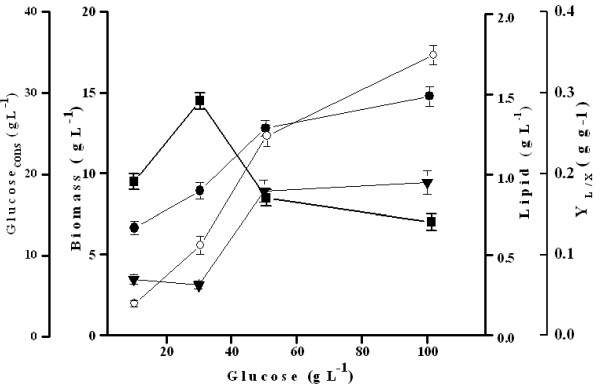
**Biomass, lipid yield, glucose consumed and lipid yield coefficients of *Y. lipolytica *NCIM 3589 grown on varying concentrations of glucose.** Biomass (g L^-1^):▾; Lipid (g L^-1^):• ; Glucose _cons_ (g L^-1^): ○; Lipid/biomass yield (g g^-1^):▪ .

Glucose concentration has a significant effect on cell growth and lipid accumulation in batch cultures (Li et al. 2007Papanikolaou et al. 2009Zhang et al. 2011). Such preliminary studies are necessary to ascertain the nutrient conditions in order to select a strain with the relevant FAME profile. Glucose is often used as a comparison basis to evaluate the performance of other carbon substrates including wastes. Therefore, we investigated the effect of initial glucose concentration, ranging from 10 to 100 g L^-1^, on biomass, SCO yields and glucose consumed (Glucose _cons_) by strain 3589. As shown in Figure 3, there was no significant substrate inhibition on cell growth of *Y. lipolytica* 3589 up to 50 g L^-1^ in 48 h. The biomass (X, g L^-1^) and lipid yield (L, g L^-1^) increased to 8.9 and 1.5 g L^-1^ for 50 g L^-1^ glucose (Figure 3). The lipid/dry biomass yield coefficient (Y_L/X_, g g^-1^) however was maximal at 30 g L^-1^ (0.29 g g^-1^) which decreased to 0.14 g g^-1^ at 100 g L^-1^ glucose. For all the initial glucose concentrations studied, significant concentrations of residual glucose remained at 48 h. (Figure 3).

### Biomass and SCO yields of *Y. lipolytica* strains on different wastes

As expected, an easily utilizable carbon source such as glucose, affected the variation in lipid yields and FAME profiles among the different *Y. lipolytica* strains. It was noted that on an optimal glucose concentration of 30 g L^-1^ in lipid accumulation media, strain 3589 exhibited a FAME profile suitable for biodiesel production. It has also been reported that addition of glucose to wastes enhances lipid production by yeast cells (Xue et al. 2008; Bialy et al. 2011). However, the fermentation cost on high glucose concentrations limits their use for biodiesel production. The use of inexpensive media for lipid fermentation is one of the possible ways to resolve this problem. Therefore, the ability of all *Y. lipolytica* strains to accumulate lipids on locally available cheap and renewable carbon sources was also evaluated. The results of preliminary studies showing biomass and lipid yields of these strains when cultivated using these wastes (1%, w/v or v/v) after 72 h are given in Table 2.

**Table 2:** **Lipid yield coefficient (Y**_**L/X**_**) of *Y*. *lipolytica *strains grown on different wastes**

**Substrate**	**NCIM 3229**	**NCIM 3450**	**NCIM 3472**	**NCIM 3589**	**NCIM 3590**
Bagasse	0.05 ± 0.02	0.06 ± 0.02	0.05 ± 0.02	0.07 ± 0.02	0.05 ± 0.01
Banana peel	0.05 ± 0.01	0.04 ± 0.01	0.06 ± 0.02	0.09 ± 0.02	0.05 ± 0.01
Cheese whey	0.03 ± 0.01	0.05 ± 0.01	0.03 ± 0.01	0.13 ± 0.01	0.05 ± 0.01
Chicken feather waste	0.04 ± 0.02	0.04 ± 0.02	0.05 ± 0.02	0.06 ± 0.03	0.03 ± 0.01
Copra meal	0.04 ± 0.01	0.03 ± 0.02	0.02 ± 0.01	0.04 ± 0.02	0.03 ± 0.01
Fish waste	0.05 ± .01	0.06 ±0.14	0.11 ± 0.14	0.14 ± 0.02	0.13 ± 0.14
Grape stalk	0.05 ± 0.02	0.04 ± 0.02	0.05 ± 0.02	0.06 ± 0.01	0.05 ± 0.02
Groundnut oil cake	0.04 ± 0.01	0.03 ± 0.01	0.02 ± 0.01	0.04 ± 0.01	0.03 ± 0.02
Groundnut shell waste	0.02 ± 0.01	0.04 ± 0.003	0.01 ± 0.002	0.03 ± 0.003	0.03 ± 0.005
Orange peel	0.03 ± 0.01	0.02 ± 0.02	0.03 ± 0.01	0.02 ± 0.02	0.03 ± 0.01
Orange pulp waste	0.06 ± 0.01	0.05 ± 0.02	0.05 ± 0.02	0.07 ± 0.02	0.07 ± 0.06
Peapod	0.03 ± 0.01	0.04 ± 0.04	0.04 ± 0.04	0.04 ± 0.02	0.04 ± 0.04
Prawn shell waste	0.03 ± 0.01	0.02 ± 0.01	0.03 ± 0.01	0.04 ± 0.02	0.03 ± 0.01
Waste cooking oil(WCO)	0.33 ± 0.2	0.45 ± 0.24	0.33 ± 0.14	0.24 ± 0.02	0.2 ± 0.24
Waste motor oil (WMO)	0.22 ± 0.06	0.55 ± 0.2	0.17 ± 0.2	0.21 ± 0.04	0.28 ± 0.2

Values are means of three independent sets of experiments.

Differences were considered statistically significant for p < 0.05.

Amongst all the locally available inexpensive wastes studied, the strains exhibited maximal lipid yield coefficients (Y _L/X_) on 4 wastes *viz*., WCO, whey, WMO and fish waste (Table 2). For the other wastes, though a good biomass was achieved, the yields of lipid obtained were relatively lower and hence were not considered further. When grown on WCO, the yields obtained were 0.33, 0.45, 0.33, 0.24 and 0.2 g g^-1^ while on WMO, the yields obtained were: 0.22, 0.55, 0.17, 0.21 and 0.28 g g^-1^, respectively for strains 3229, 3450, 3472, 3589 and 3590. Cheese whey yielded a Y _L/X_ of 0.13 g g^-1^ for strain 3589 while on fish waste, yields were 0.14 and 0.13 g g^−1^ for strains 3589 and 3590, respectively. Thus strain 3589 was able to accumulate SCO to varying degrees on all these inexpensive substrates. To date, to the best of our knowledge, no known reports on the substrates chosen in the present study for SCO production by *Y*. *lipolytica* exist other than WCO (Bialy et al. 2011).

#### Fatty acid profiles of transesterified SCOs grown on wastes

As the fatty acid composition and degree of unsaturation varies with growth substrates, the effect of these four wastes on content and type of fatty acid for all the strains was investigated. As seen in Table 3 strains 3229, 3472 and 3589 exhibited a good lipid content on WCO with respect to SFA, MUFA and PUFA. For the other wastes either the Y _L/X_ yields were low or the content of SFA, MUFA or PUFA were not desirable for biodiesel. The FAME profiles obtained were completely different to those when grown on glucose (Table 4). Though strain 3229 showed good yields, it exhibited very low content of the desirable stearic (C18:0) palmitoleic (C16:1) and oleic (C18:1) acids and hence was not considered as a suitable feedstock for biodiesel. Strain 3472 exhibited good profile when grown on WCO with a high content of MUFA (oleic acid, C18:1), reasonable amount of SFA (lignoceric acid, C24:0) and negligible amounts of PUFA. This was in complete contrast to the profile observed when grown on glucose wherein high contents of PUFAs were obtained. Strain 3589 also exhibited a reasonable profile with a high content of desirable SFA (C8:0 and C16:0) and MUFA (C18:1) with a low PUFA content (C18:2).

**Table 3:** **Biomass, lipid content, yield coefficients and fatty acid composition of *Y. lipolytica *strains on wastes**

***Y. lipolytica *strain**	**Waste used**	**X (gL**^**-1**^**)**	**L (gL**^**-1**^**)**	**Y**_**L/X**_**(gg**^**-1**^**)**	**Fatty acids (wt %)**		
					**T**_**SFA**_	**T**_**MUFA**_	**T**_**PUFA**_
NCIM 3229	Fish	8.59	0.44	0.05	52.61	34.1	13.3
	WCO	7.0	2.33	0.33	56.28	31.33	11.77
	Whey	5.91	0.23	0.03	68.4	15.6	15.5
	WMO	1.92	0.42	0.22	85.85	ND	14.13
NCIM 3450	Fish	8.57	0.53	0.06	47.6	40.8	11.46
	WCO	5.43	2.45	0.45	79.54	14.64	4.15
	Whey	6.91	0.29	0.05	97.97	0.45	1.54
	WMO	0.59	0.32	0.55	70.27	21.05	8.63
NCIM 3472	Fish	8.92	0.98	0.11	65.94	0.81	33.21
	WCO	7.98	2.67	0.33	28.04	71.94	ND
	Whey	5.7	0.2	0.04	59.6	12.63	27.74
	WMO	2.19	0.38	0.17	90.39	ND	9.59
NCIM 3589	Fish	2.86	0.39	0.14	29.57	10.35	60.03
	WCO	5.04	1.19	0.24	56.82	32.5	11.98
	Whey	2.6	0.33	0.13	61.98	3.69	34.3
	WMO	2.27	0.48	0.21	41.95	6.46	51.57
NCIM 3590	Fish	9.67	1.33	0.13	70.74	1.48	27.7
	WCO	7.65	2.2	0.28	77.12	1.2	20.54
	Whey	5.49	0.28	0.05	39.68	53.22	7.05
	WMO	1.6	0.34	0.2	73.39	ND	26.57

X: Biomass; L: lipid yield; Y _L/X_: lipid yield coefficient per gram biomass; ND: Not detected.

T _SFA_: Total of Saturated Fatty Acids; T _MUFA_: Total of Monounsaturated Fatty Acids; T_PUFA_: Total of Polyunsaturated Fatty Acids.

**Table 4:** **Fatty acid methyl ester profiles of *Y*. *lipolytica *NCIM 3472 and NCIM 3589 on WCO**

**% of fatty acid methyl ester**	**NCIM 3472**	**NCIM 3589**
Caprylic acid (C8:0)	ND	25
Lauric acid (C12:0)	ND	3.20
Myristic acid (C14:0)	ND	1.73
Palmitic acid (C16:0)	ND	21.13
Stearic acid (C18:0)	ND	3.43
Heneicosanoic acid (C21:0)	ND	1.79
Behenic acid (C22:0)	1.54	ND
Lignoceric acid (C24:0)	26.5	ND
Palmitoleic acid (C16:1)	ND	0.91
Oleic acid (C18:1n9c)	71.94	21.01
cis-11Eicosanoic acid (C20:1)	ND	2.00
cis-10-Heptadecanoic acid (C17:1)	ND	8.01
Linoleic acid (C18:2n6c)	ND	11.77
Total of fatty acids: Saturated	28.04	56.28
Total of fatty acids: Monounsaturated	71.94	31.93
Total of fatty acids: Polyunsaturated	ND	11.77
Total of fatty acids	99.9	99.98

ND: Not detected.

The values are the means of three independent determinations.

#### Effect of waste cooking oil (WCO) concentration on SCO yields

**Figure 4:**
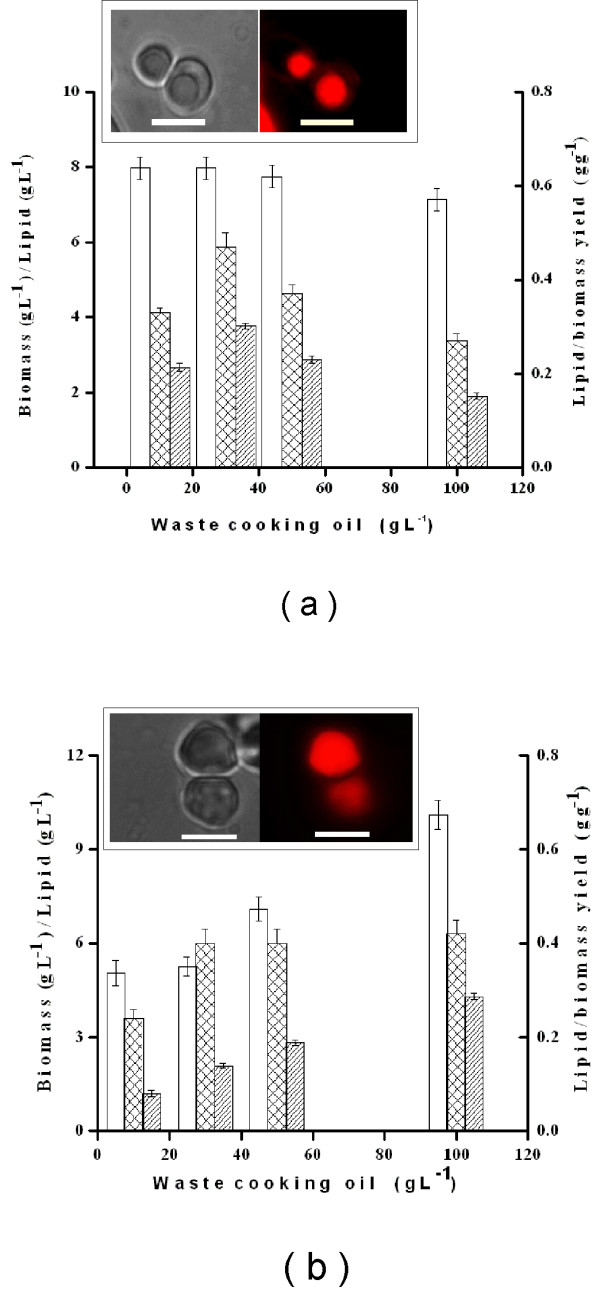
**Biomass, lipid yield and lipid yield coefficients of *Y. lipolytica *NCIM 3472 and NCIM 3589 grown on varying concentrations of WCO.** Bar (empty): Biomass (g L^-1^), Bar (oblique line): Lipid (g L^-1^), Bar (checkered): Lipid/biomass yield coefficient (g g^-1^). Inset In each graph light microscopy (left panel) and Nile red fluorescence microscopy (right panel) images of the respective *Y. lipolytica* strains under 100× oil immersion objective. Bar indicates 10 μm.

The effect of WCO concentrations, ranging from 10 to 100 g L^-1^ on biomass and SCO yields by strains 3472 and 3589 were performed in shake flasks. The WCO was composed of C16:0 (40.5%), C18:1 (40.9%) and C18:2 (10.35%), as determined by GC-FID. As shown in Figure 4a, for strain 3472, lipid accumulation could be seen by light microscopy and Nile red fluorescence (inset). Maximal biomass (7.9 g L^-1^), lipid yield (3.8 g L^-1^) and Y_L/X_ (0.47 g g^-1^) were observed at 30 g L^-1^ in 72 h. Higher WCO concentrations were inhibitory for both growth and lipid yield. For *Y. lipolytica* 3589, lipid accumulation is shown in Figure 4b inset. No significant substrate inhibition of WCO on cell growth and lipid content up to 100 g L^-1^ in 72 h was noted (Figure 4 b). The cellular biomass as total dry weight (X in g L^-1^) and lipid yield (L in g L^-1^) increased up to 10.1 and 4.3 g L^-1^ for 100 g L^-1^ WCO. The lipid/dry biomass yield coefficient (Y_L/X_, g g^-1^) was maximal at 100 g L^-1^ (0.43 g g^-1^). Hence the SCOs of both 3472 and 3589 were further investigated for their biodiesel properties.

### Biodiesel properties of transesterified SCO (FAME) from strains NCIM 3472 and NCIM 3589

Direct measurement of fuel properties of biodiesel is quite complex with high cost, error in reproducibility and requiring a considerable amount of fuel sample (Tong et al. 2011). Therefore, prediction models and mathematical equations have been developed to predict biodiesel properties from FAME composition (Azam et al. 2005; Demirbas 1998; Gunstone et al. 2007; Knothe and Steidley 2011; Lapuerta et al. 2010; Pratas et al. 2011; Tong et al. 2011). In the present study, the different physicochemical biodiesel properties *viz.*, density, kinematic viscosity, CN, SN, IV, HHV, TAN, FFA, water content of the sample, copper strip corrosion test were determined for transesterified SCO of the yeast strains 3472 and 3589. The results for the physicochemical properties summarized in Table 5 were performed experimentally as well as determined using models and/or equations based on FAME profiles. While the biodiesel properties for strain 3589 were ascertained when grown on glucose as well as WCO media those for strain 3472 were carried out for the strain grown on WCO. Biodiesel properties of 3472 were not determined when grown on glucose as the strain contained a high amount of PUFA and negligible MUFA (Table 1) which is undesirable for biodiesel.

**Table 5:** **Fuel properties of biodiesel from *Y. lipolytica *grown on glucose and WCO**

**Property/Test**	**Strain NCIM 3589 on glucose**	**Strain NCIM 3589 on WCO**	**Strain NCIM 3472 on WCO**	**US biodiesel standards ASTM D6751**	**European biodieselstandards EN14214**	**Indian biodiesel standards IS15607**
Visual test	+	+	+	NS	+	NS
Density (g cm ^-3^)*	0.81 (0.87)	1.04(0.87)	1.19 (0.87)	NS	0.8600-0.900	0.8600-0.900
Water content (vol %)*	ND	ND	ND	0.05max	0.25max	0.03max
TAN (mg NaOH/g)*	0.2	2.8	2.3	0.8max	0.5max	0.5max
FFA (%)*	0.1	1.4	1.15	NS	NS	NS
Cu strip corrosion*	Class 1a	Class 1a	Class 1a	Class 3max	Class 1max	Class 1max
CN***	56.6	50.8	59	47-65	51 min	51 min
Kinematic viscosity (40°C; mm^2^/s) ***	4.44	3.6	6.44	1.9-6.0	3.5-5.0	3.5-5.0
SN*	190.81 (194.48)	256.16 (249.4)	168.5 (177)	NS	NS	NS
IV*	65.7 (70.64)	37.8 (47.9)	54.5 (61)	NS	120max	NS
HHV(M J kg^-1^)***	40.39	36.77	41.25	NS	NS	NS
Concentration of γ-linolenic acid (C18:3) (%)*	0.1	0	0	NS	12max	NS
FAME having ≥4 double bonds (%)*	ND	ND	ND	NS	1 max	NS

WCO – waste cooking oil. The experimental values are means of three independent determinations.

ND: Not Detected; NS: Not Specified. * Experimentally determined values, followed by predicted ones, if any, in brackets.

** Calculated using experimental SN and IV while the values in brackets determined from predicted ones.

*** Predicted values as mentioned in Materials and Methods.

Among physical properties of biodiesel, strain 3589 with glucose as the carbon source, the density was predicted to be 0.87 g cm^-3^ and experimentally determined as 0.81 g cm^-3^ while viscosity was predicted to be 4.44 mm^2^ s^-1^. IV, SN and HHV are three important chemical properties of biodiesel attributed to the fatty acid profile. The IV is a crude measure of degree of unsaturation of the biodiesel and is often used in connection with its oxidative stability. The SN indicates the amount of TAG present in total lipid and HHV depends upon both IV and SN. Therefore, in the present study the SN and IV were experimentally determined as well as calculated empirically from fatty ester composition of transesterified total lipids. The experimentally determined (65.7) and the predicted (70.64) IVs were below the EN 14214 specification (120 max) and suggest good oxidative stability of the transesterified oils from strain 3589. The calculated and experimentally determined SNs were found to be 194.48 and 190.81, respectively while HHV was estimated to be 40.39 MJ kg ^-1^.

For biodiesel, CN has been found to increase with an increasing weight percentage of saturated and long chain fatty ester. In fact, methyl esters of stearic acid (C18:0), which is of relevance to biodiesel, have been found to possess the highest CN (> 80) (Knothe 2008). In the present study, methyl esters of long chain saturated fatty acids namely stearic acid (C18:0) and palmitic acid (C16:0) were also present in the transesterified yeast oil. The calculated CN was found to be 56.6 (Table 5), and within the range suggested by the standard norms. The TAN and FFA content as determined experimentally according to EN14214 were estimated to be 0.2 mg KOH g^-1^ and 0.1%, in accordance with the biodiesel standards. Other chemical properties of biodiesel evaluated were the concentration of linolenic acid (C18:3) and wt % of FAMEs having ≥ 4 double bonds. From the fatty acid profile of the yeast SCO, it can be seen that the concentration of C18:3 (0.1%) was well below the specified limit of 12 max and fatty esters with ≥ 4 double bonds were not detected in the transesterified oil (Table 5). These values are in the acceptable range of international biodiesel standard norms suggesting the possible suitability of biodiesel from strain 3589 when grown on glucose.

When grown on WCO as substrate, for strain 3589, the density and TAN were found to be slightly higher at 1.04 g cm^-3^ and 2.8 mg g ^-1^ NaOH, respectively. For strain 3472, the density (1.19 g cm^-3^), TAN (2.3 mg g^-1^ NaOH), CN (59) and kinematic viscosity (6.44 mm^2^ s^-1^) were found to be much higher than the recommended limits given by the international biodiesel standard norms. All other values for the SCOs from strains 3472 and 3589 were found to lie within the specified limits of the biodiesel standards (Table 5). This is in fact the first report on characterization of biodiesel from any *Y. lipolytica* strain.

## Discussion

In this study, SCO from strains of a known lipid accumulating model organism *Y. lipolytica* were evaluated to select a lipid yielding strain with higher level of saturated and monounsaturated FAMEs for biodiesel production. All five *Y. lipolytica* strains tested *viz.* NCIM 3229, 3450, 3472 3589 and 3590 were able to accumulate > 20% of their biomass as cellular lipids.

Glucose concentration has previously been shown to influence the yields of lipid produced, composition of fatty acids as well as their degree of unsaturation in oleaginous yeasts *Rhodotorula glacialis* DBVPG 4785 (Amaretti et al., 2010) *Candida* sp. 107 (Gill et al. 1997) and *Trichsporon fermentans* (Zhu et al., 2008). A comparison with other *Y. lipolytica* strains grown on glucose has been tabulated and shown in Table 6. Thus, in this study, the strains produced biomass (3.77 - 7.42 g L^-1^) and lipid yields, (0.84 - 1.48 g L^−1^) in 30 g L^-1^ glucose in a short fermentation time of 48 h. While it can be seen that the biomass obtained was comparable to earlier studies, the *Y. lipolytica* strains used in this study produced higher lipid yield coefficients (0.22-0.31 g g^-1^ biomass) to those reported in literature (0.04- 0.14 g g^-1^ biomass) (Table 6). Amongst the lipids, neutral lipids comprising of triacylglycerol (TAG) are the most suitable for direct conversion to biodiesel. In all the five strains the neutral lipid fraction was found to be 88-89% (w/w) of the total lipid content. Since biodiesel is derived by trans-esterifying the SCO, the fatty acid composition of the original feedstock determines the quality of biodiesel. Methyl esters from MUFAs *viz.,* palmitoleic (C16:1) and oleic (C18:1) acids are warranted as they are liquid at room temperature and would help in good flow properties. The percentage of unsaturated fatty acids affects the oxidative stability of the final product and quality of biofuel during extended storage (Knothe 2005). A high total PUFA content results in increased viscosity, which again is undesirable for biodiesel. Thus, an ideal biodiesel is made mainly from methyl esters of both SFA and MUFA with low PUFA (Ramos et al. 2009). In this study, amongst all the 5 strains studied, SCO from *Y. lipolytica* 3589 cultured on 30 g L^-1^ glucose in 48 h exhibited a good yield coefficient (Y_L/X_) (0.29 g g^-1^), biomass (4.16 g L^−1^), high neutral lipid fraction (89% w/w of total lipid) and a desirable fatty acid profile containing palmitic (24.1%), stearic (7.7%), oleic (38.6%), palmitoleic (11%) acids with a lower content of unwanted PUFAs, a composition essential for good quality biodiesel. Strains 3229, 3450, 3472 and 3590 either contained high amounts of PUFA or were low in MUFAs when grown on glucose, making them unsuitable for biodiesel.

**Table 6:** **Comparison of biomass, lipid and fatty acid profiles of various strains of *Y*. *lipolytica *reported on glucose**

**S No.**	**Strain of *Y. lipolytica***	**Glucose**	**Time**	**X**	**L**	**Glu**_**cons**_	**Y**_**L/X**_	**Fatty acids (%)**			**References**
		**(g L**^**-1**^**)**	**(h)**	**(gL**^**-1**^**)**	**(gL**^**-1**^**)**	**(gL**^**-1**^**)**	**(gg**^**-1**^**)**	**T**_**SFA**_	**T**_**MUFA**_	**T**_**PUFA**_	
1	LGAM S (7) 1	28	47.5	5	0.35	22.5	0.07	17.2	53.1	10.4	Papanikolaou et al. 2006
2	W 29 (ATCC 20460)	20	24	-	-	-	0.05	11.94	30.46	47.43	Beopoulos et al. 2008
3	ACA-YC-5028	30	98	5.5	-	28.2	-	20.5	67.3	12.1	Papanikolaou et al. 2009
	ACA-YC-5029		119	4.9	0.02	23.9	0.04	17.3	75.4	7.3	
	ACA-YC-5030		119	5.9	-	28.9	-	16.4	78.3	5.3	
	ACA-YC-5031		72	5.6	-	16.9	-	-	-	-	
	ACA-YC-5032		96	5.1	-	13.5	-	-	-	-	
	ACA-YC-5033		94	5.1	0.02	24.8	0.05	22.6	66.3	11.1	
	LFMB 15		95	5.2	-	13.2	-	-	-	-	
	W 29		142	5.8	0.06	29.2	0.06	20.4	62.3	17.3	
	ACA-YC-5029	60	219	3.9	0.01	50.8	0.1	17.4	72.3	9.1	
	ACA-YC-5033		309	5.5	0.02	58.8	0.14	20.4	64.5	11.2	
	W 29		315	5	0.03	57.7	0.07	22.4	59.5	18.1	
4	W 29 (ATCC 20460)	35	72	5.6	0.6	13.8	0.11	20.3	61.2	17	Sarris et al. 2011
	ACA-YC-5028		72	-	-	-	-	25.6	50.4	24	
	ACA-YC-5033		24	4.2	0.5	5.4	0.12	23.3	62.4	12.1	
5	NCIM 3229	30	96	7.42	0.83	21.11	0.22	70.6	0.6	28.8	Present study
	NCIM 3450		24	3.23	1.01	8.36	0.31	4.9	22.8	72.4	
	NCIM 3472		48	3.58	0.89	14.02	0.25	17.1	0	82.4	
	NCIM 3589		48	3.10	0.9	11.29	0.29	34.6	50.1	15.1	
	NCIM 3590		24	1.05	0.3	7.94	0.28	64.1	14.3	21.6	

-: Not mentioned; X: Biomass; L: lipid yield; Glu _cons:_ Glucose consumed; Y _L/X_: lipid yield coefficient per gram biomass; T _SFA_: Total of Saturated Fatty Acids; T _MUFA_: Total of Monounsaturated Fatty Acids; T_PUFA_: Total of Polyunsaturated Fatty Acids.

The physico-chemical properties of FAMEs of strain 3589 grown on glucose were comparable with the predicted/estimated values reported for biodiesel obtained from other oleaginous yeasts (Liu and Zhao 2007; Thiru et al. 2011). These values lie within the acceptable range of international biodiesel standard norms and were comparable to vegetable oils (Leung et al. 2010). The free fatty acid (FFA) content was found to be below 0.1% and FAMEs ≥ 4 double bonds were not detected, well within the specifications of EN 14214.

*Y. lipolytica* strains, can induce a notable accumulation of reserve lipid when grown on hydrocarbons and fatty substrates. Based on the lipid yield coefficients and FAME profiles, it was seen that the strains 3472 and 3589 could effectively utilize WCO as an inexpensive waste substrate for SCO production. An earlier report by Bialy et al. 2011, has indicated that growth of *Y. lipolytica* resulted in an increase in total lipid content (0.58 g g^-1^) when grown on frying vegetable oil waste (0.5%, w/v) supplemented with 10 g L^-1^ glucose. Recently an oleaginous yeast *Y. lipolytica* has been shown to produce SCO (0.59 g g^-1^) on sugarcane bagasse hydrolysate medium (Tsigie et al. 2011) while Zygomycetous fungi like *Mortierella isabellina* have been shown to produce significant quantities of biomass (23.1 g L^-1^) and Y_L/X_ (0.17 g g^-1^) when grown on cheese whey (100%, v/v) (Vamvakaki et al. 2010). A previous study has reported a lipid yield coefficient of 0.44-0.54 g g^-1^ on stearin by *Y. lipolytica* ACA-DC-50109 (Papanikolaou and Aggelis 2010) while ATCC 20460 yielded 0.20 g g ^-1^ on biodiesel-derived glycerol (Andre et al. 2009). Thus, lipid yield coefficients obtained on WCO in the present study are promising as preliminary data indicates that strain 3589 was able to utilize WCO up to 100 g L^-1^ with Y _L/X_ of 0.43 g g^-1^ while strain 3472 exhibited a Y _L/X_ of 0.47 g g^-1^ on 30 g L^-1^ WCO with higher concentrations being inhibitory.

Both strains exhibited a different FAME profile when grown on WCO as compared to glucose which was reflected in their biodiesel properties. While the TAN values were higher in both the strains than the expected norms, all other properties for strain 3589 seemed to lie within the specified range. For strain 3472, the CN value, density and kinematic viscosity were also found to be much higher than the expected range. This would most likely be due to the high content (26.5%) of the long chain SFA (C24:0) present in the strain. Generally long chain SFAs are not desirable for biodiesel as they are known to increase viscosity thereby affecting flow properties (Knothe 2005). The fuel properties for strain 3589 could be further improved on WCO supplemented with glucose during optimization as reported in the case of *Y. lipolytica* (Bialy et al. 2011) and *Trichosporon fermentans* (Zhu et al., 2008). This approach may not be possible for strain 3472 as it leads to increased levels of PUFAs when grown on glucose.

Toxic compounds such as aldehydes, semialdehydes, hydrocarbons, alkoxy radicals and acids are generated during the process of reheating WCO which can be inhibitory for microbial growth (Kulkarni and Dalai 2006). Strain 3589 could grow up to 100 g L ^-1^ WCO and accumulate 0.43 g g^-1^ lipid indicating the ability of the strain to tolerate toxic compounds and accumulate lipids at high WCO concentrations. On the other hand, high concentrations of WCO were found to be inhibitory for growth and lipid accumulation in strain 3472 which showed a steady decrease in Y _L/X_ from 10–100 g L ^-1^ (0.33-0.27 g g ^-1^), making it unsuitable for further optimization and scale-up studies.

The results obtained in the present study were comparable with those reported for other yeasts. For example, a study on *Cryptococcus curvatus* for biodiesel production reported a number of fuel properties with similar values including acid value (0.47), density (0.879 g cm^-3^) and iodine value (59) (Thiru et al. 2011). Previously, Liu and Zhao (2007) predicted CN values of 59.9 and 63.5 for two oleaginous yeasts, *Lipomyces starkeyi* and *Rhodosporidium toruloides*, respectively.

Thus, SCO from the tropical marine yeast *Y. lipolytica* 3589 seems to be a potential feedstock for biodiesel production with the neutral lipid fraction as the major component of their total lipids, the presence of higher quantities of saturated and monounsaturated C16 and C18 fatty acids and lower concentration of long chain PUFAs as the major features. The strain could accumulate lipids upto 0.43 g g^-1^ in the presence of 100 g L^-1^ WCO. The experimentally determined and predicted biodiesel properties based on FAME composition of the yeast SCO of strain 3589 grown on glucose and on WCO are found to lie within the range specified by international biodiesel standard specifications and was therefore identified as a promising strain for further studies. This is the first report on the physico-chemical characterization of biodiesel from any *Y. lipolytica* strain.

## Authors' contributions

GK performed the experiments as a part of her doctoral work while CJ carried out the experiments on the wastes. MK participated in the statistical analysis of the data and SSZ helped to draft the manuscript. All this work was carried out under the supervision of ARK who conceived and coordinated the study, designed the experiments and drafted the manuscript. All the authors have read and approved the final manuscript.

## Competing interests

The authors declare that they have no competing interests.
